# Safety and Efficacy of Rechallenge With Immune Checkpoint Inhibitors in Advanced Solid Tumor: A Systematic Review and Meta‐Analysis

**DOI:** 10.1002/cam4.70324

**Published:** 2024-10-28

**Authors:** Huijun Xu, Yang Yang, Ying Yan, Mengge Li, Shusheng Wu, Lulu Cao, Wenju Chen, Huiqin Luo, Yifu He

**Affiliations:** ^1^ Division of Life Sciences and Medicine, Department of Oncology, the First Affiliated Hospital of USTC University of Science and Technology of China Hefei Anhui China; ^2^ Division of Life Sciences and Medicine, Department of Ultrasound, the First Affiliated Hospital of USTC University of Science and Technology of China Hefei Anhui China

**Keywords:** immune checkpoint inhibitors, immune‐related adverse events, rechallenge, solid tumor

## Abstract

**Background:**

Immune checkpoint inhibitors (ICIs) have drastically shifted the current landscape toward a wide variety of malignancies. However, ICIs are interrupted owing immune‐related adverse events (irAEs), therapy completion, and disease progression. The risk–benefit of rechallenged ICIs remains inconclusive. Herein, a systematic review and meta‐analysis were conducted to evaluate the safety and efficacy of ICI rechallenge in the treatment of advanced solid tumor.

**Methods:**

PubMed, Web of Science, Embase, and Cochrane Library were searched to analyze the efficacy and safety of ICI rechallenge. The study protocol was approved by the PROSPERO International Register of Systematic Reviews (CRD42022372222). The last updated search date was March 2, 2024. Objective response rate (ORR), disease control rate (DCR), overall survival (OS), and incidence rates of all‐ and high‐grade irAEs were evaluated.

**Results:**

A total of 41 retrospective studies comprising 2343 patients were ultimately enrolled for qualitative and quantitative assessments. A total of 1200 (51.2%) individuals were male and the median age was 66 years (range 18–97 years). The majority of the tumors was lung cancer (*n* = 898, 38.3%). The occurrence rates of all‐grade and high‐grade (grade 3 or 4) irAEs between initial and readministration ICIs were not significantly different (all‐grade: OR, 0.75, 95% CI: 0.39–1.45, *p* = 0.40; *I* ^2^ = 87%; high‐grade: OR, 0.96, 95% CI: 0.62–1.49, *p* = 0.87, *I* ^2^ = 65%). ICIs restart presented a decreased ORR and DCR compared to initial ICI administration (ORR: OR, 0.36, 95% CI: 0.23–0.56, *p* < 0.00001; *I* ^2^ = 67%; DCR: OR, 0.62, 95% CI: 0.43–0.89, *p* = 0.010; *I* ^2^ = 53%). Seven studies with 513 patients for survival analysis revealed a nonsignificant difference in OS between the ICIs rechallenge and discontinuation cohorts (hazard ratio [HR]: 0.68, 95% confidence interval (CI): 0.35 to 1.35, *p* = 0.27).

**Conclusion:**

Rechallenging immunotherapy is feasible, and patients should be carefully evaluated by a multidisciplinary team prior to initial therapy for close monitoring and assessment of the risk–benefit ratio. Therefore, prospective trials are essential to guide clinicians in the decision‐making process.

**PROSPERO** **Registration:** CRD42022372222.

## Graphical abstract

Our study provides a significant contribution to the field of oncology, particularly in understanding the nuanced impact of immune checkpoint inhibitors (ICIs) on patient outcomes after rechallenge. Our study is relevant to Cancer Medicine, as it answers a crucial question in immunotherapy and offers practical insights for clinical decision‐making through a comprehensive review of existing evidence. Our detailed data synthesis and statistical analyses will benefit clinicians and researchers in navigating ICI treatment complexities. Our manuscript adds to the discourse on immunotherapy and lays the foundation for future research.

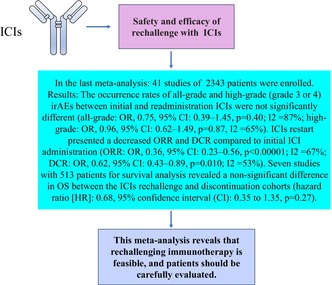

## 1 Introduction

In the last decade, compared to conventional therapies, immune checkpoint inhibitors (ICIs) have drastically shifted the current landscape across numerous cancers. Immunotherapy agents such as ICIs focus on inhibiting key proteins such as programmed cell death ligand 1 (PD‐L1), programmed cell death protein 1 (PD‐1), and cytotoxic T lymphocyte antigen 4 (CTLA‐4), which play critical roles in regulating immune responses against tumors and have contributed to promising progress in improving patients survival benefits [1, 2, 3]. ICIs play a pivotal role in T‐cell activation by interrupting of the negative effects of T‐cell inhibition, which reinvigorates antitumor immunity and protects against tumor cell immune evasion [4, 5].

Unfortunately, the pursuit of successful immunotherapy is marked by an increase in immune‐related adverse events (irAEs). IrAEs have been reported to be positively associated with prognosis [6]. IrAEs vary across almost all organ systems, from mild to severe symptoms and are life‐threatening [7, 8, 9, 10, 11]. A large proportion of patients with tumors must discontinue ICI treatment because of irAEs [12]. Furthermore, interruption of ICI treatment includes tumor progression and completion of a fixed course. As the safety and efficacy of ICI readministration are not well established, the efficacy of ICI retreatment remains inconclusive.

We performed a comprehensive review and meta‐analysis to evaluate the safety and efficacy of ICI rechallenge in advanced solid tumor. We present the following article according to the Preferred Reporting Items for Systematic Reviews and Meta‐Analyses Reporting Checklist.

### 1.1 Methods

Embase, Web of Science, PubMed, and Cochrane Library have served as sources for identifying pertinent research published to date. The last updated search date was March 2, 2024. The study protocol was approved by the PROSPERO International Register of Systematic Reviews (CRD42022372222). To formulate the search strategy for our study on oncological treatments, we focused on identifying critical terms and their corresponding medical subject headings (MeSHs). These terms encompassed a broad range of descriptors including “neoplasm” and “immune checkpoint inhibitors.” Additionally, the strategy incorporated specific names for ICIs such as “ipilimumab, nivolumab, durvalumab, pembrolizumab, avelumab, cemiplimab, atezolizumab, tremelimumab, and ticilimumab.” To explore the concept of therapeutic readministration, we also included keywords associated with “rechallenge,” like “retreat, readminister, restart, reinitiate, resume, and reinduction.” This comprehensive selection of terms was designed to ensure a thorough review of the existing literature and to facilitate a nuanced understanding of treatment dynamics in the field of oncology. Retrieval terms were combined using Boolean operators (OR, NOT, and AND). The search terms were linked using Boolean logic (AND, OR, NOT) as outlined in Table S1, detailing the search strategy.

### 1.2 Inclusion and Exclusion Criteria

Following the initial review of titles and abstracts, two reviewers (HJX and YY) carefully assessed all the studies for final inclusion. HJX and YY were considered when there was a discrepancy. Conferences, abstracts, case reports, and animal studies were also excluded. We followed the population‐intervention‐comparator‐outcome study design (PICOS) framework to meticulously craft our research question and guide the associated literature review. A population with advanced solid tumors is of interest. The intervention of interest was rechallenged with immunotherapy, mainly included PD1, PD‐L1, and CTLA4 inhibitors. The outcomes of interest included irAEs, time interval from AE to ICI therapy, objective response rate (ORR), disease control rate (DCR), and overall survival (OS) after the initial treatment and retreatment. Detailed data were obtained from the original study, Supporting Information, and e‐mail to the corresponding author.

### 1.3 Quality Assessment

Independent researchers HJX and YY evaluated the methodological integrity of all included studies using the Newcastle‐Ottawa Scale (NOS) criteria [13]. NOS criteria included subject selection (0–4), comparability (0–2), and clinical outcomes (0–3). The scores ranged from 0 (low‐quality) to 9 (high‐quality). NOS scores ≧ 4 were considered high‐quality, according to the modified Jada scale [14].

### 1.4 Data Synthesis and Statistical Analysis

The National Cancer Institute (Bethesda, MD, USA) stipulates In version 5 of the Common Terminology Criteria for Adverse Events, the National Cancer Institute (Bethesda, MD, USA) stipulates that irAEs classified as grade 3 or higher are considered high‐grade. ORR measures both complete and partial response rates, whereas DCR measures complete response, partial response, and stable disease rates. OS was defined as the period from the start of ICI therapy to death.

For data synthesis, Review Manager 5.3 (Cochrane Community, London, UK) was used for graphical representation. The aggregation of odds ratios (OR) and their 95% confidence intervals (CI) was calculated using the Mantel–Haenszel (M‐H) method [15]. *I* ^2^ statistics were used to estimate the heterogeneity. When substantial heterogeneity was detected, we utilized the random‐effects model; otherwise, a fixed‐effects model was implemented.

## 2 Results

**FIGURE 1:**
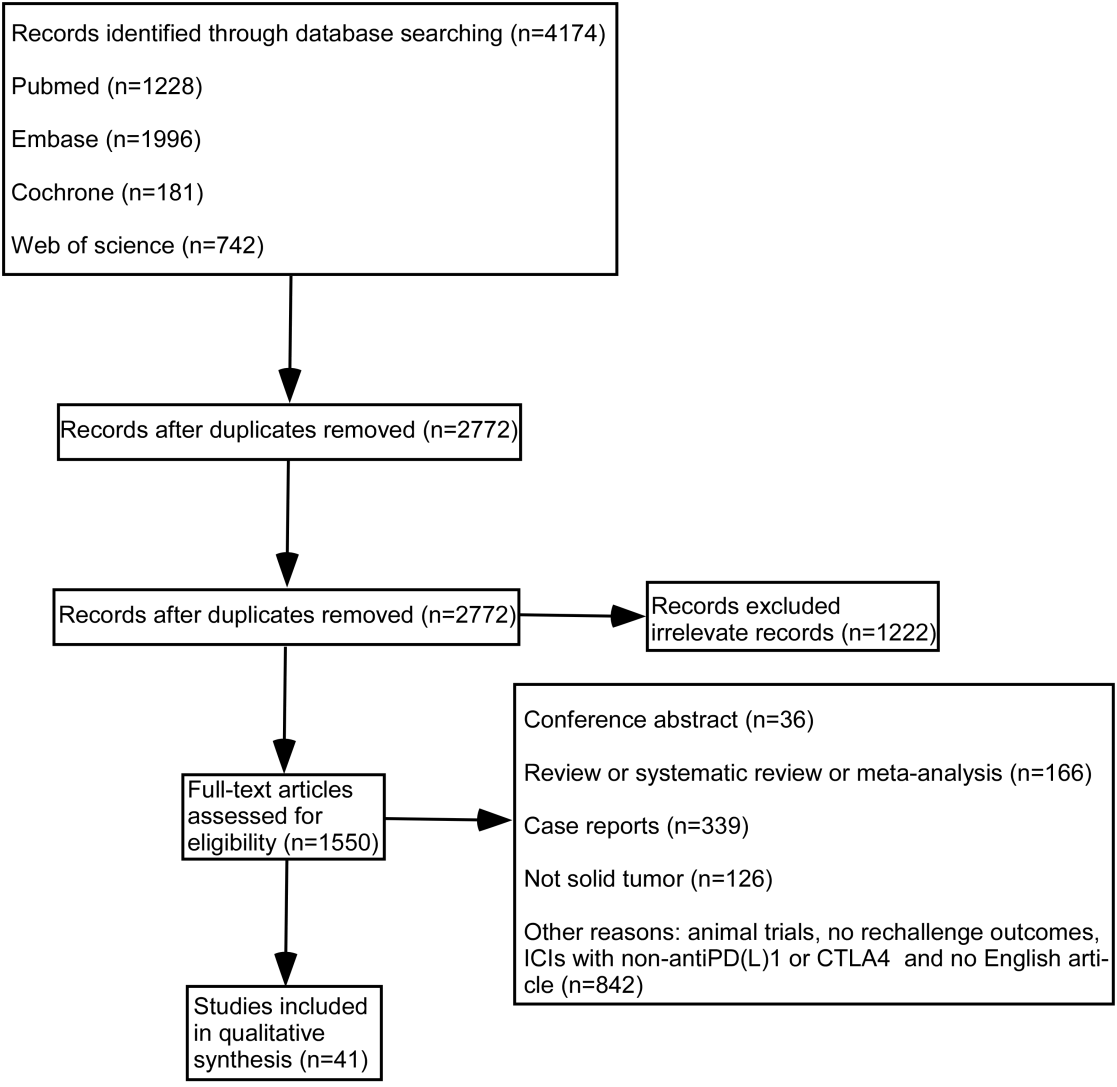
PRISMA 2020 flow diagram. “*n*” represents the numbers of studies.

We identified 4174 articles using a database search. After removing duplicate and ineligible articles, 41 retrospective studies comprising 2343 patients were ultimately included in the assessment. Figure 1 shows the detailed retrieval procedure. The score of each study was more than 7, indicating a high quality according to the NOS (Table S2). Table 1 presents the attributes of the patients included in the meta‐analysis. A total of 1200 (51.2%) individuals were male and the median age was 66 years (range 18–97 years). The most common tumor types were lung cancer (*n* = 898, 38.3%), followed by melanoma (*n* = 707, 30.2%), and genitourinary cancer (*n* = 488, 20.8%). The remaining patients had head and neck cancer (*n* = 91, 3.9%), hepatic/biliary tract cancer (*n* = 61, 2.6%), gynecological cancer (*n* = 41, 1.7%), Merkel cell carcinoma (*n* = 29, 1.2%), gastrointestinal cancer (*n* = 2, 0.1%), and other cancers (*n* = 26, 1.1%). In the administration of ICIs, the primary therapeutic strategies include monotherapy or combined application of anti‐PD1/PD‐L1 and anti‐CTLA4 agents. The median time interval between ICI administration and irAEs in the initial and rechallenge cohorts was 62 and 54 days, respectively. The median PFS in the prior and restarted immunotherapy groups was 6.4 and 4.3 months, respectively.

**TABLE 1:** Summary of involved studies characteristics.

First author, year	Cancer type	PD‐L1 expression (%, number)	Number of patients	ICI type	DCR	ORR	mPFS (m)	Total irAEs	High‐grade irAEs	Reasons for ICI discontinuation	Median interval time to initial irAEs (*d*)	Number of patients	ICI type	DCR	ORR	mPFS (m)	mOS (m)	Total irAEs	High‐grade irAEs	Median interval time to rechallenge irAEs (*d*)
Niki 2018 [18]	NSCLC	≥ 5: 3	11	Anti‐PD1	7/11	5/11	4.9	5/11	0/11	NE	NE	11	Anti‐PD1	5/11	3/11	2.7	NE	5/11	0/11	NE
Kartolo 2021 [17]	Multiple	NE	85	Anti‐CTLA4 plus PDL1	NE	NE	NE	85/85	21/85	irAEs	62	40	Anti‐CTLA4 plus PDL1	NE	NE	NE	21.9	31/40	7/40	41
Alaiwi 2020 [16]	mRCC	NE	80	Anti‐PD (L) 1, Anti‐PD (L) 1 plus CTLA4	NE	25/80	NE	80/499	43/499	irAEs	84	36	Anti‐PD (L) 1, anti‐PD (L) 1 plus CTLA4	6/36	6/36	NE	NE	18/36	7/36	84
Asher 2019 [35]	Melanoma	NE	19	Anti‐CTLA4 plus PD (L) 1	15/19	11/19	NE	NE	NE	irAEs	127	7	NE	6/7	4/7	NE	NE	NE	NE	NE
Fujisaki 2021 [20]	NSCLC	≥ 50: 19, 1–49: 3, < 1: 2	52	Anti‐PD1	36/52	24/52	14.3	93/231	25/231	irAEs	NE	14	Anti‐PD1	14/14	10/14	15.3	NR	6/14	3/14	NE
Fujita 2019 [37]	NSCLC	≥ 50: 9, 1–49: 3, < 1: 3, unknow: 3	18	Anti‐PD1	14/18	7/18	NE	NE	NE	NE	NE	18	Anti‐PDL1	7/18	0	NE	NE	NE	NE	NE
Fujita 2020 [38]	NSCLC	≥ 50: 0, 1–49: 5, < 1: 5, unknow: 5	15	Anti‐PDL1	5/15	0	NE	NE	NE	PD	NE	15	Anti‐PD1	4/15	0	NE	NE	NE	NE	NE
Gobbini 2020 [34]	NSCLC	< 1: 9, ≥ 1: 43, ≥ 25: 22, ≥ 50: 21	144	Anti‐PD (L) 1	109/144	71/144	13	NE	27/144	PD, irAEs, clinical decision	NE	140	Anti‐PD (L) 1	68/140	23/140	4.4	18	24/140	7/140	NE
Guo 2022 [39]	NSCLC	≥ 50: 35, 1%–50%: 14, < 1: 27, unknow: 23	99	Anti‐PD (L) 1, CTLA4	NE	74/99	NE	NE	NE	irAEs	NE	40	Anti‐PD (L) 1, CTLA4	NE	2/40	NE	NE	NE	NE	66
Hepner 2021 [21]	Melanoma	NE	47	Anti‐CTLA4/PD1	47/47	37/47	11	44/47	18/47	irAEs	NE	47	Anti‐CTLA4, PD1	21/47	12/47	5	17	27/47	18/47	NE
Isik 2021 [50]	Multiple	median: 55, IQR 5–80	50	Anti‐PD (L) 1, CTLA4	NE	NE	NE	37/2143	NE	irAEs	NE	16	Anti‐PD (L) 1, CTLA4	NE	NE	NE	NE	NE	NE	NE
Katayama 2020 [40]	NSCLC	≥ 50: 14, 1–49: 8, < 1: 7, unknow: 6	35	Anti‐PD (L) 1	24/35	12/35	4	NE	NE	NE	NE	35	Anti‐PD (L) 1	15/35	1/35	2.7	7.5	NE	NE	NE
Koch 2022 [23]	Uveal melanoma	NE	177	Anti‐CTLA4, PD1	50/177	16/177	NE	74/177	44/177	Resistance and toxicity	NE	52	Anti‐CTLA4, PD1	10/52	5/52	NE	NE	21/52	10/52	NE
Lee 2021 [49]	Multiple	NE	27	Anti‐PD (L) 1, CTLA4	17/27	13/27	NE	NE	NE	irAEs	52.5	15	Anti‐PD (L) 1, CTLA4	11/15	NE	NE	NE	NE	NE	72
Li 2020 [41]	Melanoma	NE	102	Anti‐PD (L) 1, CTLA4	68/102	48/102	NE	NE	NE	irAEs	NE	31	Anti‐PD (L) 1, CTLA4	23/31	8/31	NE	NE	NE	NE	NE
Mouri 2019 [24]	Lung cancer	NE	49	Anti‐PD1	NE	23/49	NE	49/187	15/49	irAEs	76	21	Anti‐PD1	18/21	3/21	NE	NE	15/21	1/21	NE
Nomura 2017 [42]	Melanoma	NE	8	Anti‐PD1	5/8	1/8	4.1	NE	NE	PD	NE	8	Anti‐PD1	5/8	2/8	4.3	NE	NE	NE	NE
Patrinely Jr 2021 [6]	Multiple	NE	164	Anti‐PD (L) 1, CTLA4	125/164	101/164	NE	NE	NE	irAEs	52	66	Anti‐PD (L) 1, CTLA4	NE	NE	NE	NE	17/66	7/66	32
Hasson 2021 [7]	Multiple	NE	7	Anti‐PD (L) 1	NE	NE	NE	NE	NE	irAEs	NE	3	Anti‐PD (L) 1	NE	NE	NE	NE	NE	NE	NE
Ravi 2020 [26]	Renal cell carcinoma	NE	69	Anti‐PD (L) 1, CTLA4	54/69	25/69	8.2	49/69	18/69	PD, irAEs, other	NE	69	Anti‐PD (L) 1, CTLA4	41/69	15/69	5.7	NE	31/69	11/69	NE
Santini 2018 [27]	NSCLC	NE	68	Anti‐PD (L) 1, CTLA4	NE	30/68	NE	68/482	33/482	irAEs	71	38	Anti‐PD (L) 1	NE	18/38	NE	NE	20/38	8/38	32
Siddiqui 2021 [29]	Multiple	NE	231	Anti‐PD (L) 1, CTLA4	NE	NE	NE	231/231	17/231	AE, complete, PD, lost of follow‐up, therapy ongoing	56	61	Anti‐PD (L) 1, CTLA4	46/61	22/61	NE	NE	46/61	14/61	NE
Stege 2020 [30]	Merkel Cell carcinoma	NE	8	Anti‐PD (L) 1	5/8	4/8	NE	4/8	1/8	PD, irAEs	NE	8	Anti‐PD (L) 1	5/8	5/8	NE	NE	3/8	0	NE
Stege 2021 [43]	Merkel Cell carcinoma	NE	20	Anti‐PD (L) 1	20/20	17/20	NE	NE	NE	irAEs, patient preference, CR, PD	NE	8	Anti‐PD (L) 1	8/8	7/8	NE	NE	NE	NE	NE
Takahara 2022 [31]	NSCLC	low: 12, high: 11, unknown: 1	24	Anti‐PD (L) 1	11/24	2/24	NE	9/24	4/24	PD, other	NE	24	Anti‐PD (L) 1	NE	NE	NE	NE	4/24	3/24	NE
Tikkanen 2020 [44]	Multiple	NE	106	Anti‐PD (L) 1	39/106	15/106	NE	NE	NE	irAEs, CR, complete	NE	8	Anti‐PD (L) 1	3/8	1/8	NE	NE	NE	NE	NE
Tsui 2021 [5]	Multiple	NE	81	Anti‐PD (L) 1, CTLA4	NE	NE	NE	81/81	NE	irAEs	NE	44	Anti‐PD (L) 1, CTLA4	NE	NE	NE	NE	NE	NE	NE
Watanabe 2019 [33]	NSCLC	≥ 50: 7, 1–49: 1, < 1: 4, unknown: 2	14	Anti‐PD (L) 1	8/14	3/14	3.7	9/14	3/14^a^	NE	NE	14	Anti‐PD (L) 1	3/14	1/14	1.65	NE	5/14	0*	NE
eill 2021 [9]	Multiple	NE	20	Anti‐PD (L) 1, CTLA4	NE	NE	NE	NE	NE	irAEs	41	9	Anti‐PD (L) 1, CTLA4	7/9	6/9	NE	NE	NE	NE	NE
Yamagata 2021 [45]	NSCLC	≥ 50: 10	27	Anti‐PD (L) 1	NE	14/27	NE	NE	NE	irAEs	57	3	Anti‐PD (L) 1	2/3	0	NE	NE	NE	NE	NE
Yang 2022 [46]	NSCLC	1 <: 8, 1–49: 21, ≥ 50: 16, unknown: 36	81	Anti‐PD (L) 1	44/81	18/81	NE	NE	NE	PD, irAEs	NE	45	Anti‐PD (L) 1	32/45	1/45	3.2	NE	NE	NE	NE
Fujita 2018 [36]	NSCLC	≥ 50: 6, 1–49: 1, < 1: 0	12	AntiPD1	9/12	7/12	6.2	NE	NE	NE	NE	12	Anti‐PD1	5/12	1/12	3.1	NE	NE	NE	NE
Bila 2024 [10]	HNSCC	NE	19	ICI	NE	NE	NE	19/19	8/19	irAEs	NE	3	NE	NE	NE	NE	NE	NE	NE	NE
Feng 2024 [19]	NSCLC	≥ : 18, 1–49: 9, < 1: 20, unknown: 64	111	Anti‐PD (L)1, CTLA4	92/111	46/111	6.6	50/111	15/111	NE	NE	111	Anti‐PD (L) 1, CTLA4	79/111	19/111	5.9	NE	42/111	20/111	NE
Kim 2023 [22]	Gynecologic cancers	≥ 1: 16, < 1: 2, unknown: 2	20	AntiPD1	9/20	5/20	2.8	8/20	4/20	PD, irAEs, other	NE	20	Anti‐PD1	8/20	3/20	1.8	21.3	4/20	3/20	NE
Li 2023 [11]	Cervical cancer	NE	15	AntiPD1	NE	NE	7	NE	NE	irAEs	NE	15	Anti‐PD1	NE	NE	3	8	12/15	6/15	NE
Makrakis 2023 [47]	Urothelial carcinoma	NE	25	Anti‐PD (L) 1, CTLA4	22/25	18/25	NE	NE	NE	PD, irAEs	NE	25	Anti‐PD (L) 1, CTLA4	16/25	8/25	NE	NE	NE	NE	NE
Nardin 2023 [25]	Melanoma	NE	85	AntiPD1, CTLA4	NE	NE	NE	43/85	19/85	Disease control	NE	85	Anti‐PD (L) 1	NE	NE	21	NR	28/85	10/85	NE
Nizam 2024 [48]	Urothelial carcinoma	NE	16	Anti‐PD (L) 1	15/16	8/16	NE	NE	NE	irAEs	NE	10	Anti‐PD (L) 1	7/10	2/10	15.5	18.9	NE	NE	NE
Scheiner 2023 [28]	Hepatocellular carcinoma	NE	58	Anti‐PD (L) 1	34/58	13/58	NE	31/58	9/58	PD, irAEs, other	NE	58	Anti‐PD (L) 1	32/58	15/58	NE	NE	28/58	10/58	NE
Ueno 2024 [32]	Esophageal cancer	NE	44	Anti‐PD1	NE	NE	NE	44/116	6/116	irAEs	72	14	Anti‐PD1	NE	NE	NE	NE	14/14	0	NE

Abbreviations: CTLA4, cytotoxic T lymphocyte antigen 4; DCR, disease control rate; High‐grade, grade ≥ 3 was defined as high‐grade; HNSCC, head and neck squamous cell carcinoma; ICI, immune checkpoint inhibitor; irAEs, immune‐related adverse events; m, month; mOS, median overall survival; mPFS, median progression‐free survival; NE, not evaluable; NSCLC, nonsmall cell lung cancer; ORR, objective response rate; PD1, programmed cell death protein 1; PD‐L1, programmed cell death ligand 1.

^a^ Was referred to grade ≥ 2.

### 2.1 Safety

**FIGURE 2:**
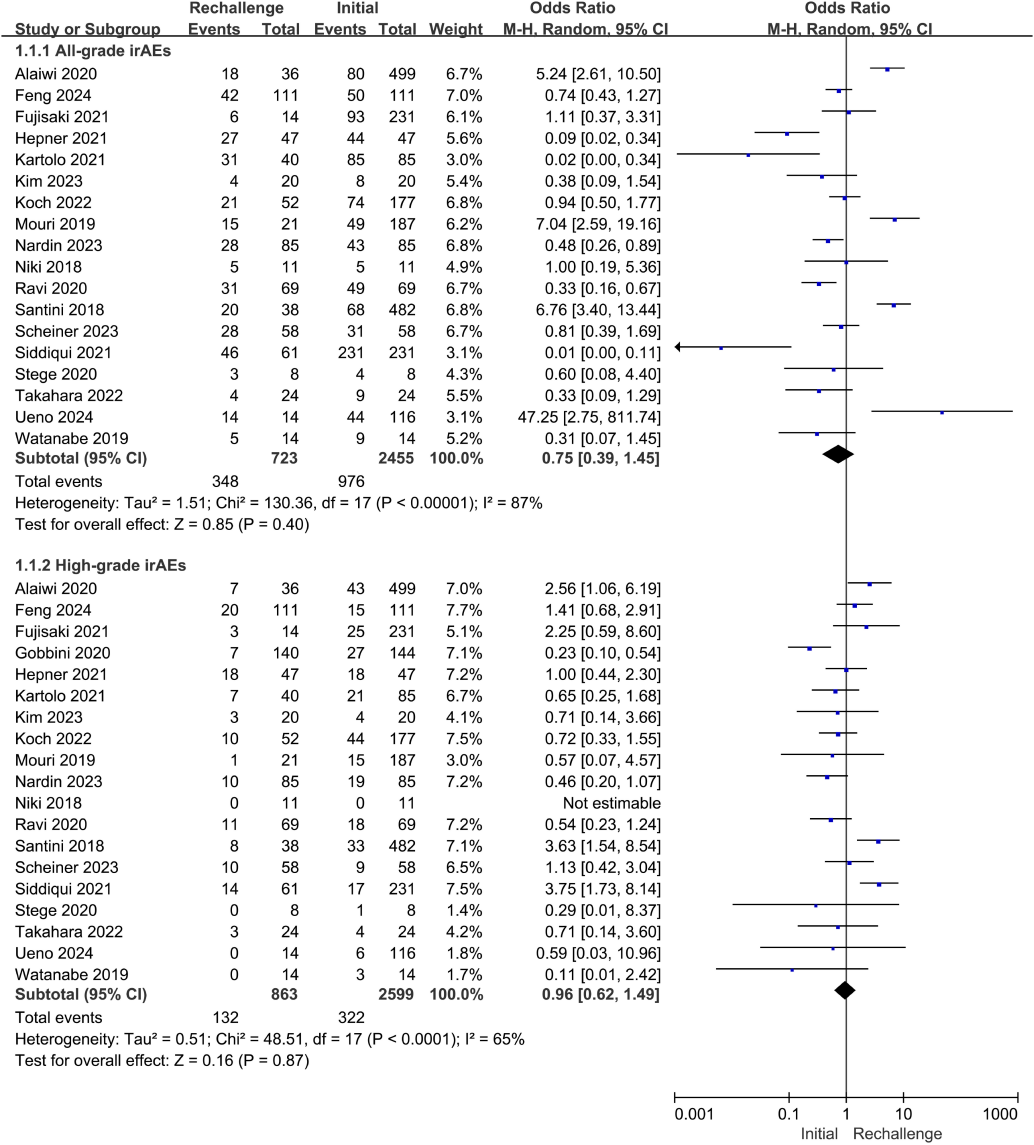
Forest plot (random‐effects model) of the associations between ICIs rechallenge and the incidence of all‐grade or high‐grade irAEs. The sizes of the squares mean the weight of the study; High‐grade was defined as grade > =3. ICIs, immune checkpoint inhibitors; irAEs, immune‐related adverse events; M‐H, Mantel–Haenszel model; CI, confidence. interval.

There were 19 studies in the analysis of safety [16, 17, 18, 19, 20, 21, 22, 23, 24, 25, 26, 27, 28, 29, 30, 31, 32, 33, 34]. The pooled rates of all‐ and high‐grade irAEs in the overall treatment were 41.7% and 13.1%, respectively. The recurrence rates of all‐ and high‐grade irAEs in the ICI rechallenge group were 34.3% and 15.3%, respectively. Fortunately, the occurrence rates of all‐grade and severe‐grade (grade 3 or 4) irAEs between prior ICIs treatment and readministration did not differ significantly (all‐grade: OR, 0.75, 95% CI: 0.39–1.45, *p* = 0.40; *I* ^2^ = 87%; high‐grade: OR, 0.96, 95% CI: 0.62–1.49, *p* = 0.87, *I* ^2^ = 65%) (Figure 2). Egger's tests showed no publication biases (all‐grade: *p* = 0.324; high‐grade: *p* = 0.348).

We further performed a subset analysis for the same ICI type for the initial and rechallenge administrations, such as antiPD1 to antiPD1. The occurrence of ICIs, both all‐grade and high‐grade, exhibited no significant differences between the two treatment modalities (all‐grade adverse events: OR 2.15, 95% CI 0.54–8.56, *p* = 0.28, heterogeneity *I* ^2^ = 78%; high‐grade adverse events: OR 1.00, 95% CI 0.41–2.4, *p* = 1.00, *I* ^2^ = 0%) (Figures S1 and S2).

### 2.2 Efficacy

**FIGURE 3:**
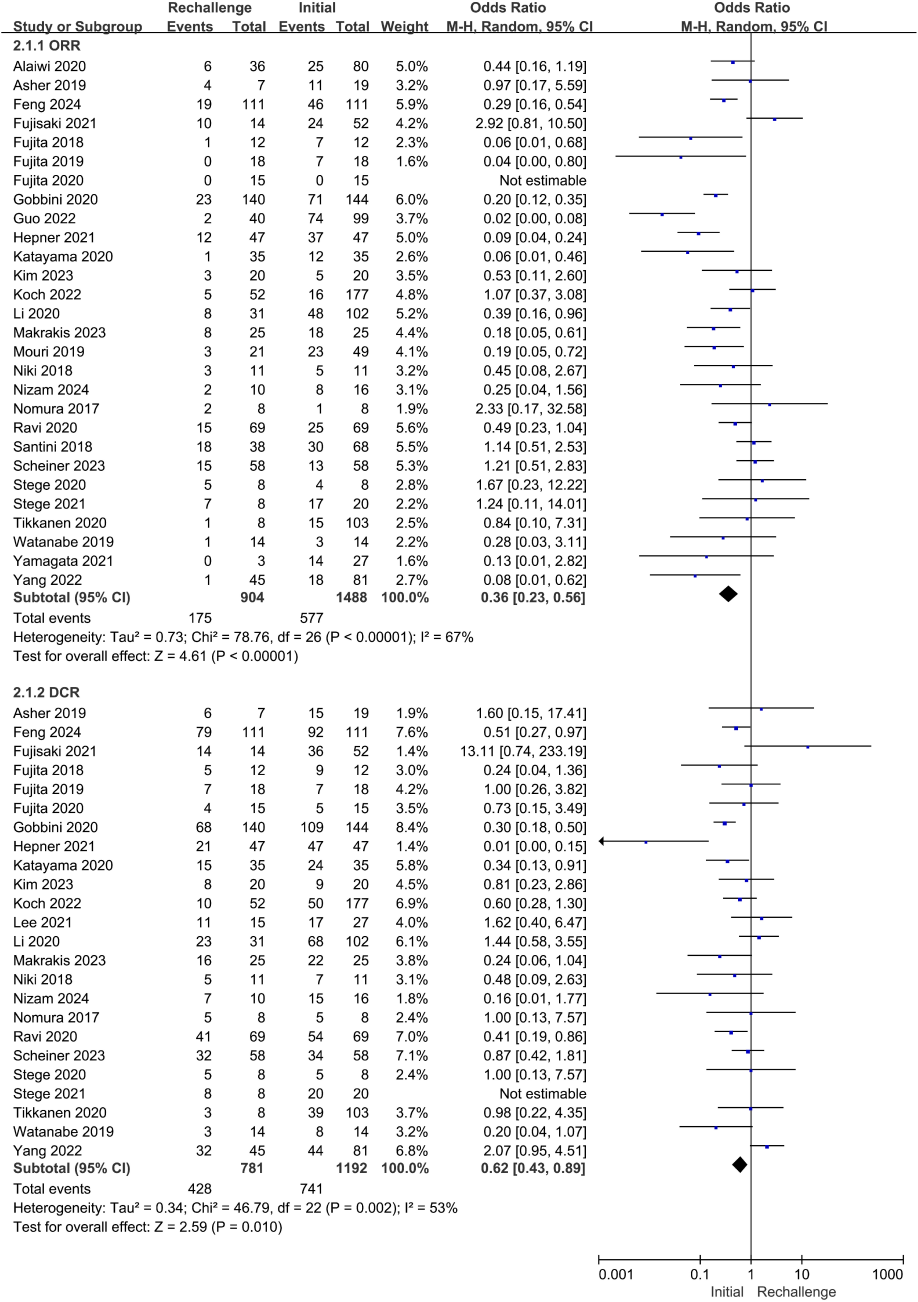
Forest plot (random‐effects model) of the associations between ICIs rechallenge and ORR or DCR. The sizes of the squares mean the weight of the study. ICIs, immune checkpoint inhibitors; ORR, objective response rate; DCR, disease control rate; M‐H, Mantel–Haenszel model; CI, confidence interval.

A total of 29 cohort studies were included in the efficacy analysis [16, 18, 19, 20, 21, 22, 23, 24, 26, 27, 28, 30, 33, 34, 35, 36, 37, 38, 39, 40, 41, 42, 43, 44, 45, 46, 47, 48, 49]. The combined ORR and DCR for ICIs throughout the treatment period were 31.4% and 59.2%, respectively. The recurrence rates of ORR and DCR after immunotherapy resumption were 19.4% and 54.8%, respectively. However, ICI restart presented a decreased ORR compared to the initial ICI administration (OR, 0.36, 95% CI: 0.23–0.56, *p* < 0.00001; *I* ^2^ = 67%). Egger's tests proved no evidence of publication biases (ORR: *p* = 0.804; DCR: *p* = 0.740). In the rechallenge group, DCR notably decreased compared with previous ICI treatments, exhibiting an odds ratio of 0.62 with a confidence interval of 95% ranging from 0.43 to 0.89 and a statistical significance of *p* = 0.010; heterogeneity was moderate at *I* ^2^ = 53% (Figure 3).

The efficacy of the same ICIs between the initial treatment and retreatment treatment was further analyzed. The results did not show a difference in ORR for the same ICIs between the two subgroups (OR, 0.54; 95% CI: 0.17–1.70, *p* = 0.29; *I* ^2^ = 62%). Unexpectedly, the rechallenged cohort achieved a reduced ORR, compared to the initial group, when switching the ICIs types (OR, 0.97; 95% CI: 0.49–1.91, *p* = 0.03; *I* ^2^: not applicable) (Figure S3). Likewise, DCR was not distinct for the same and different ICIs between the two subgroups (the same ICIs: OR, 0.97; 95% CI: 0.49–1.91, *p* = 0.93; *I* ^2^ = 37%; the different ICIs: OR, 0.87; 95% CI: 0.32–2.42, *p* = 0.80; *I* ^2^ = 0%) (Figure S4).

**FIGURE 4:**
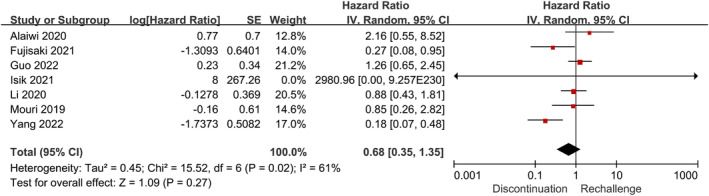
Forest plot (random‐effects model) for overall survival. The sizes of the squares mean the weight of the study. The diamond indicates the pooled hazard ratio and 95% Cl. CI, confidence interval.

Moreover, seven studies with 513 patients identified prognosis for research [16, 20, 24, 39, 41, 46, 50]. A meta‐analysis of the studies compared the OS between ICIs rechallenge and discontinuation populations. However, the results revealed a nonsignificant difference in the OS between the two groups. The weighted HR of all seven studies for OS was 0.68, with a 95% confidence interval ranging from 0.35 to 1.35 (*p* = 0.27) (Figure 4).

## 3 Discussion

In the last decade, ICIs have transformed cancer treatment by targeting and eliminating tumor cells using the immune system. In real‐world, it is not well‐established whether restart ICIs treatment after discontinuation, due to adverse events, tumor progression, and treatment completion. Our study provides a significant contribution to the field of oncology, particularly in understanding the nuanced impact of ICIs on patient outcomes after rechallenge. Our study conducted a meta‐analysis of ICI rechallenge safety and efficacy in patients with advanced solid tumors. Currently, the main data on rechallenge with ICIs are derived from published series with high heterogeneity. This systematic review summarizes up‐to‐date evidence regarding the efficacy and safety of ICIs rechallenge for advanced solid tumors. In this study, the efficacy of ICIs readministration for ORR of 19.4% and DCR of 54.8% achieved a certain degree of benefit, although lower than the initiation of ICIs, which is consistent with previous studies [19, 47, 51]. Possible reasons include posterior‐line therapy, poor Eastern Cooperative Oncology Group (ECOG) scores and advanced patient’ conditions when resuming ICI therapy. Zhao et al. found no difference in ORR and DCR between the two cohorts in solid tumor [52]. In the subgroup analysis of NSCLC, efficacy was consistent with that of solid tumor [52]. However, a previous study showed a lower ORR (17.1%) and DCR (71.2%) in ICI rechallenge than in the initial treatment (ORR: 41.4%; DCR: 82.3%) for NSCLC [19]. Hepner et al. reported similar results in melanoma. The enrolled 47 patients study showed that 37 and 12 patients had ORR, and 47 and 21 patients had DCR in the initial and rechallenged treatments, respectively [21].

Owing to the lack of available data, there is a paucity of current literature on meta‐analysis of survival. Only one Italian team performed meta‐analysis of OS between resumption and interruption of immunotherapy [53]. In the analysis of the four studies, the rechallenge cohort showed better survival. A similarly prolonged OS was identified in a retrospective study involving 428 advanced NSCLC patients [27]. Unexpectedly, our study achieved the opposite results. In our analysis, the OS of patients rechallenged with ICIs was lower than that of patients who discontinued treatment. Fujisaki observed that a better prognosis was associated with severe irAEs and readministration of ICIs [20]. Therefore, clinicians should be cautious when rechallenging patients using ICIs.

Strikingly, our data indicated that neither the all‐grade nor high‐grade irAE rates in the rechallenge group differed, which is consistent with previous studies [19, 51]. Feng et al. published a retrospective study of 111 patients with advanced NSCLC. The all‐grade rates in the rechallenged and initial cohort were 37.8% and 45%, respectively. Grade 3 or higher irAEs occurred in 13.5% and 18.0% of patients in the rechallenge and initial treatment groups, respectively. Further statistical analysis revealed no differences in all‐grade and high‐grade irAEs between the two groups [19]. A study on melanoma indicated that irAEs of any grade irAEs were higher in the initial treatment group than in the rechallenge group. However, high‐grade irAE rates were similar in the initial and rechallenged cohort [21]. This suggests that rechallenge with ICIs may be feasible in patients with a high response rate to initial immunotherapy after irAE remission.

In summary, the risks of adverse events, especially severe irAEs, in rechallenged immunotherapy are feasible; however, restarting ICI therapy might not prolong patient’ survival. Further research is needed to improve rechallenge protocols and identify predictive markers for better patient stratification.

The strengths of this meta‐analysis are the application of prognosis estimation and the large number of enrolled 2343 patients and 41 studies. However, our study had some limitations. First, similar to previous studies, none of the reports comprising our evidence used prospective data, which might have led to selection bias and generated concerns regarding the quality of evidence. Second, the ICIs retreatment timing and regimens were based on attending doctors, and were not standardized. In addition, most data on efficacy were based on ORR and DCR rather than PFS or OS, making it difficult to perform a comprehensive analysis. Finally, because the data gathered from the studies in question were inadequate, it was difficult to identify effective markers for ICI rechallenge.

## 4 Conclusions

The main limitation of this study is its heterogeneous and sparse nature, which makes it difficult to draw a definitive conclusion regarding the rechallenge strategy. It is possible to restart immunotherapy, but a multidisciplinary team must meticulously assess patients to ensure close monitoring and evaluate the risk–benefit ratio, prior to initial therapy. Above all, it is essential to perform prospective trials to guide clinicians in the decision‐making processes. Our data synthesis and statistical analyses will benefit clinicians and researchers in navigating ICI treatment complexities. Our study adds to the discourse on immunotherapy and lays the foundation for future research.

## Author Contributions

**Huijun Xu:** conceptualization (equal), data curation (equal), formal analysis (equal), writing – original draft (equal), writing – review and editing (equal). **Yang Yang:** conceptualization (equal), formal analysis (equal), methodology (equal), writing – review and editing (equal). **Ying Yan:** formal analysis (equal), methodology (equal). **Mengge Li:** formal analysis (equal), software (equal), supervision (equal). **Shusheng Wu:** data curation (equal), validation (equal). **Lulu Cao:** data curation (equal), formal analysis (equal). **Wenju Chen:** data curation (equal), formal analysis (equal). **Huiqin Luo:** investigation (equal), methodology (equal). **Yifu He:** conceptualization (equal), funding acquisition (equal), methodology (equal).

## Conflicts of Interest

The authors declare no conflicts of interest.

## Supporting information


Figure S1.

Figure S2.

Figure S3.

Figure S4.

**Table S1.** PubMed search strategy.

Table S2.

Data S1.

## Data Availability

A Prism 2020 checklist has been created and attached.
