# Supplementary figures and images for: Safety and Efficacy of Rechallenge With Immune Checkpoint Inhibitors in Advanced Solid Tumor: A Systematic Review and Meta‐Analysis
Source: Cancer Med. 2024 Oct 28;13(20):e70324. doi: 10.1002/cam4.70324 (PMC11513547; doi:10.1002/cam4.70324)

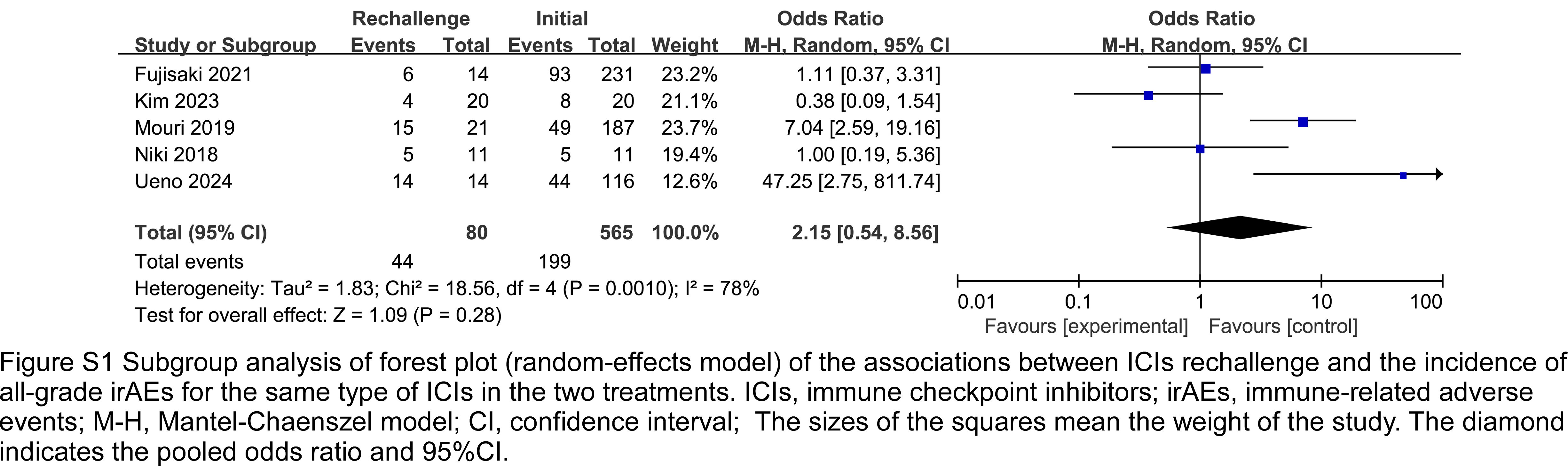

Supplement: Supplementary file 1 — Figure S1. [file CAM4-13-e70324-s006.tif]

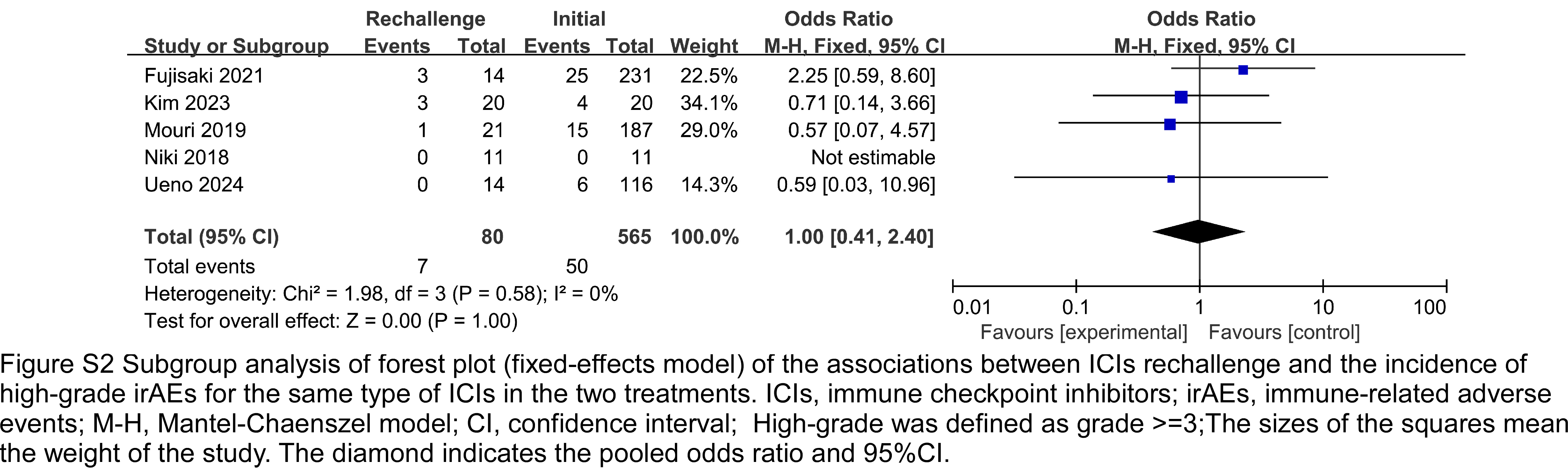

Supplement: Supplementary file 2 — Figure S2. [file CAM4-13-e70324-s003.tif]

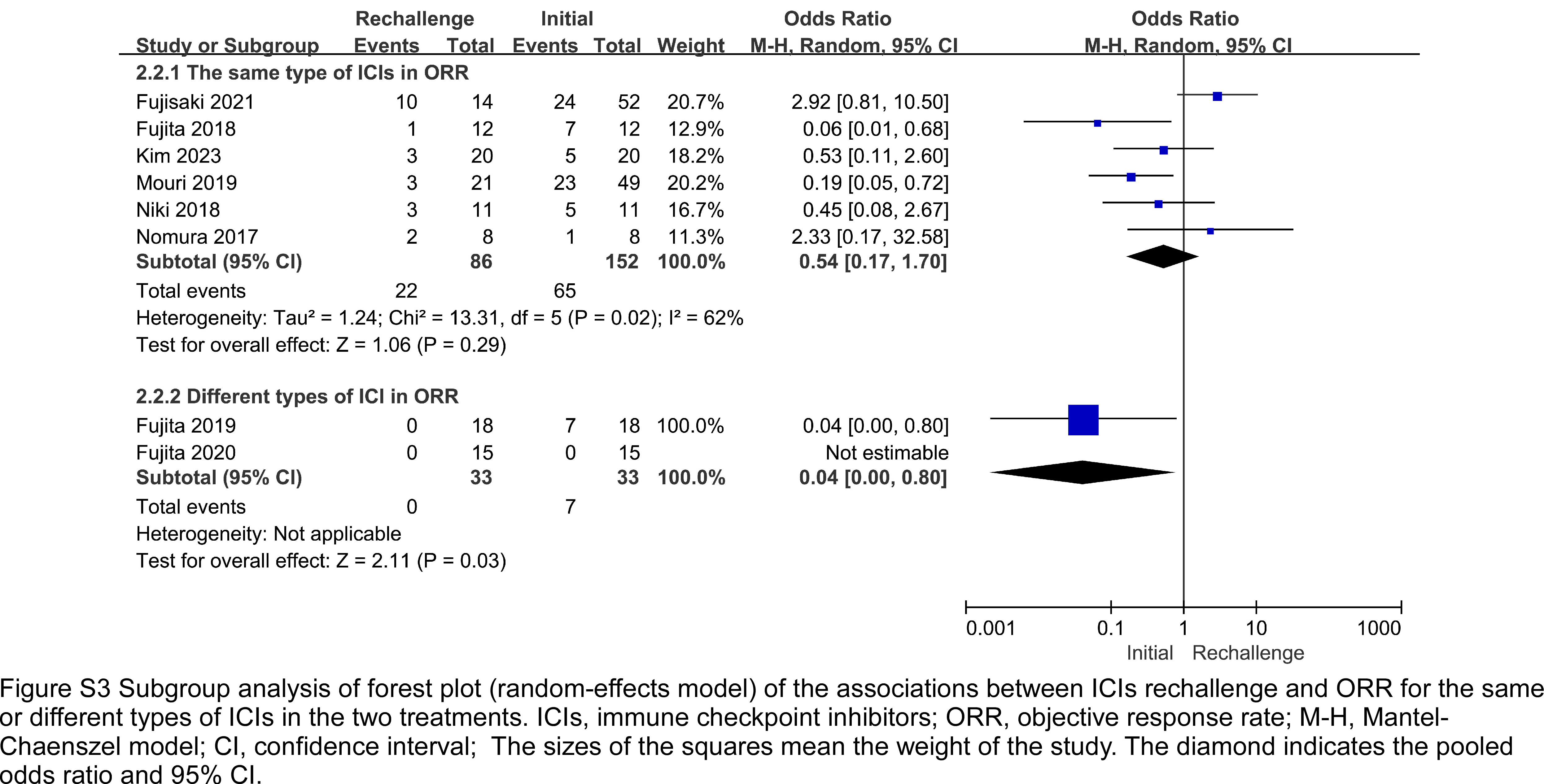

Supplement: Supplementary file 3 — Figure S3. [file CAM4-13-e70324-s004.tif]

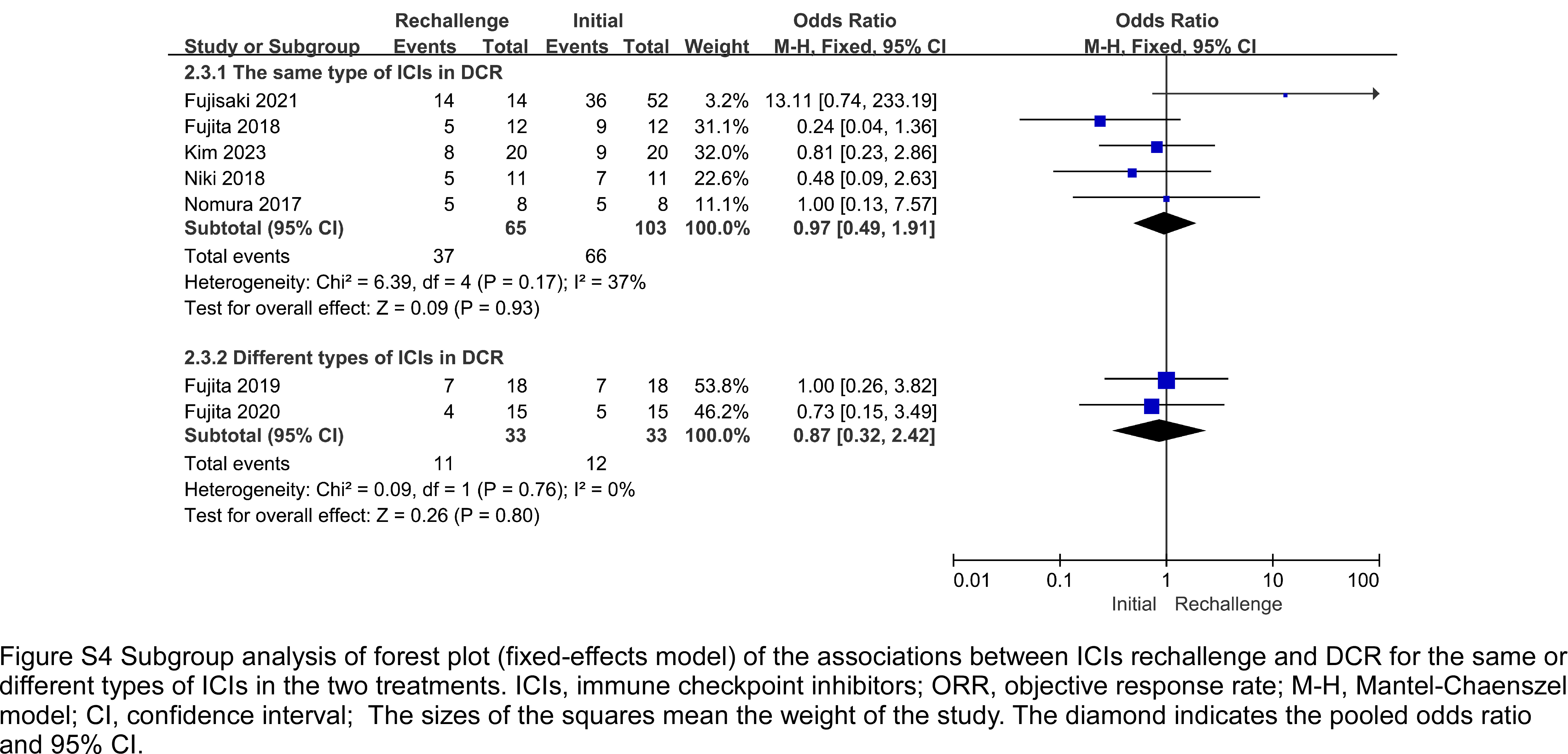

Supplement: Supplementary file 4 — Figure S4. [file CAM4-13-e70324-s001.tif]
