# Supplementary material for: Safety and Efficacy of Rechallenge With Immune Checkpoint Inhibitors in Advanced Solid Tumor: A Systematic Review and Meta‐Analysis
Source: Cancer Med. 2024 Oct 28;13(20):e70324. doi: 10.1002/cam4.70324 (PMC11513547; doi:10.1002/cam4.70324)
Supplement: Supplementary file 5 — Table S1. PubMed search strategy. [file CAM4-13-e70324-s005.doc]

**Table** S1

| Immune checkpoint inhibitors=1# | "Immune Checkpoint Inhibitors"(Mesh) OR "Checkpoint Inhibitors, Immune" OR "Immune Checkpoint Inhibitor OR Checkpoint Inhibitor, Immune" OR "Immune Checkpoint Blockers" OR "Checkpoint Blockers, Immune" OR "Immune Checkpoint Blockade" OR "Checkpoint Blockade, Immune" OR "Immune Checkpoint Inhibition" OR "Checkpoint Inhibition, Immune" OR "PD-L1 Inhibitors" OR "PD L1 Inhibitor" OR "PD-L1 Inhibitor" OR "Programmed Death-Ligand 1 Inhibitors" OR "Programmed Death Ligand 1 Inhibitors" OR "PD-1-PD-L1 Blockade" OR "Blockade, PD-1-PD-L1" OR "PD 1 PD L1 Blockade" OR "CTLA-4 Inhibitors" OR "CTLA 4 Inhibitors" OR "CTLA-4 Inhibitor" OR "CTLA 4 Inhibitor" OR "Cytotoxic T-Lymphocyte-Associated Protein 4 Inhibitors" OR "Cytotoxic T Lymphocyte Associated Protein 4 Inhibitors" OR "Cytotoxic T-Lymphocyte-Associated Protein 4 Inhibitor" OR "Cytotoxic T Lymphocyte Associated Protein 4 Inhibitor" OR "PD-1 Inhibitors" OR "PD 1 Inhibitors OR PD-1 Inhibitor" OR "Inhibitor, PD-1" OR "PD 1 Inhibitor" OR "Programmed Cell Death Protein 1 Inhibitor" OR "Programmed Cell Death Protein 1 Inhibitors" OR "nivolumab" OR "pembrolizumab" OR "atezolizumab" OR "durvalumab" OR "avelumab" OR "ipilimumab" OR "cemiplimab" OR "tremelimumab" OR "ticilimumab" |
| --- | --- |
| Rechallenge=2# | "Rechallenge" OR "retreatment" OR "readministration" OR "restart" OR "resume" OR "reinduction" OR "reinitiate" |
| Neoplasm=3# | "Neoplasm" (Mesh) OR "cancer" OR "tumor" OR "malignancy" |
|  | 1# AND 2# AND 3# |
