# Supplementary material for: Safety and Efficacy of Rechallenge With Immune Checkpoint Inhibitors in Advanced Solid Tumor: A Systematic Review and Meta‐Analysis
Source: Cancer Med. 2024 Oct 28;13(20):e70324. doi: 10.1002/cam4.70324 (PMC11513547; doi:10.1002/cam4.70324)
Supplement: Supplementary file 6 — Table S2. [file CAM4-13-e70324-s002.doc]

Table S2 Risk of Bias Quality Assessment Using the Newcastle-Ottawa Quality Assessment Scale

| Study | Selection | | | | Comparability | Outcome | | | Score |
| --- | --- | --- | --- | --- | --- | --- | --- | --- | --- |
|  | Representativeness of the exposed cohort | Selection of the nonexposed cohort | Ascertainment of exposure | Demonstration that outcome of interest was not present at start of study | Comparability of cohorts on the basis of the design or analysis | Assessment of outcome | Was follow-up long enough for outcomes to occur | Adequacy of follow-up of cohorts |  |
| Niki et al 2018 (18) | * | * | * | * | * | * | * | * | 8 |
| Kartolo et al 2021 (17) | * | * | * | * | * | * |  |  | 6 |
| Alaiwi 2020(16) | * | * | * | * | * | * |  |  | 6 |
| Asher 2019(35) | * | * | * | * | ** | * | * |  | 8 |
| Fujisaki 2021(20) | * | * | * | * | * | * | * | * | 8 |
| Fujita 2019(37) | * | * | * | * | * | * |  |  | 6 |
| Fujita 2020(38) | * | * | * | * | * | * |  |  | 6 |
| Gobbini 2020(34) | * | * | * | * | ** | * | * | * | 9 |
| Guo 2022(39) | * | * | * | * | * | * | * | * | 8 |
| Hepner 2021(21) | * | * | * | * | * | * | * | * | 8 |
| Isik 2021(50) | * | * | * | * | * | * |  | * | 7 |
| Katayama 2020(40) | * | * | * | * | * | * | * | * | 8 |
| Koch 2022(23) | * | * | * | * | * | * | * | * | 8 |
| Lee 2021(49) | * | * | * | * | * | * | * | * | 8 |
| Li 2020(41) | * | * | * | * | * | * | * |  | 7 |
| Mouri 2019(24) | * | * | * | * | * | * |  | * | 7 |
| Nomura 2017(42) | * | * | * | * | * | * | * | * | 8 |
| Patrinely Jr 2021(6) | * | * | * | * | * | * |  | * | 7 |
| Hasson 2021(7) | * | * | * | * | * | * | * | * | 8 |
| Ravi 2020(26) | * | * | * | * | * | * | * |  | 7 |
| Santini 2018(27) | * | * | * | * | * | * |  |  | 6 |
| Siddiqui 2021(29) | * | * | * | * | * | * | * |  | 7 |
| Stege 2020(30) | * | * | * | * | * | * | * |  | 7 |
| Stege 2021(43) | * | * | * | * | * | * | * |  | 7 |
| Takahara 2022(31) | * | * | * | * | * | * | * |  | 7 |
| Tikkanen 2020(44) | * | * | * | * | * | * | * |  | 7 |
| Tsui 2021(5) | * | * | * | * | * | * |  |  | 6 |
| Watanabe 2019(33) | * | * | * | * | * | * |  |  | 6 |
| Weill 2021(9) | * | * | * | * | * | * | * |  | 7 |
| Yamagata 2021(45) | * | * | * | * | * | * | * |  | 7 |
| Yang 2022(46) | * | * | * | * | * | * | * |  | 7 |
| Fujita 2018(36) | * | * | * | * | * | * |  |  | 6 |
| Bila 2024(10) | * | * | * | * | * | * |  |  | 6 |
| Feng 2024(19) | * | * | * | * | * | * | * | * | 8 |
| Kim 2023(22) | * | * | * | * | * | * | * | * | 8 |
| Li 2023(11) | * | * | * | * | * | * |  | * | 7 |
| Makrakis 2023(47) | * | * | * | * | * | * | * |  | 7 |
| Nardin 2023(25) | * | * | * | * | * | * | * | * | 8 |
| Nizam 2024(48) | * | * | * | * | * | * |  | * | 7 |
| Scheiner 2023(28) | * | * | * | * | * | * |  | * | 7 |
| Ueno 2024(32) | * | * | * | * | * | * |  | * | 7 |
